# Supplementary material for: The genomic origins of the Bronze Age Tarim Basin mummies
Source: Nature. 2021 Oct 27;599(7884):256–61. doi: 10.1038/s41586-021-04052-7 (PMC8580821; doi:10.1038/s41586-021-04052-7)
Supplement: Supplementary file 1 — This PDF file includes five sections of Supplementary text. (1) Environmental setting of Xinjiang; (2) Archaeological sites and context; (3) Linguistic background of the population history in Xinjiang; (4) Detailed description of genetic isolation of the Tarim group; and (5) Tarim mummies and the pre-pastoralist Central Asian genetic substratum. [file 41586_2021_4052_MOESM1_ESM.pdf]

nature portfolio

https://doi.org/10.1038/s41586-021-04052-7

Supplementary information

The genomic origins of the Bronze Age Tarim Basin mummies

In the format provided by the authors and unedited

The genomic origins of the Bronze Age Tarim mummies

Fan Zhang, Chao Ning, Ashley Scott, Qiaomei Fu, Rasmus Bjørn, Wenying Li, Dong Wei, Wenjun Wang, Linyuan Fan, Idilisi Abuduresule, Xingjun Hu, Qiurong Ruan, Alipujiang Niyazi, Guanghui Dong, Peng Cao, Feng Liu, Qingyan Dai, Xiaoyian Feng, Ruowei Yang, Zihua Tang, Pengcheng Ma, Chunxiang Li, Shizhu Gao, Yang Xu, Sihao Wu, Shaoqing Wen, Hong Zhu, Hui Zhou, Martine Robbeets, Vikas Kumar, Johannes Krause, Christina Warinner, Choongwon Jeong, Yinqiu Cui

This PDF file includes:

Supplementary Text

1 Environmental setting of Xinjiang …… 2
2 Archaeological sites and context …… 2
2.1 The Dzungaria Basin …… 2
2.1.1 Ayituohan …… 2
2.1.2 Songshugou …… 3
2.1.3 Nileke …… 3
2.2 The Tarim Basin …… 3
2.2.1 Xiaohe …… 4
2.2.2 Gumugou …… 5
2.2.3 Beifang …… 5
3 Linguistic background of the population history in Xinjiang …… 5
4 Detailed description of genetic isolation of the Tarim group…… 6
5 Tarim mummies and the pre-pastoralist Central Asian genetic substratum…… 7

References…… 9

Other supplementary materials for this manuscript include the following:

Data File S1. Sample information, qpAdm modeling results and phenotypic traits of the studied individuals (.xlsx)

Data File S2. Ancient and modern populations analyzed in this study (.xlsx)

Data File S3. Dairy peptides identified within the Xiaohe dental calculus samples (.xlsx)

1. Environmental setting of Xinjiang

Xinjiang is situated in the western-most part of China and has a continental dry climate and a typical mountain-oasis-desert ecosystem. It is among the world's harshest inhabited terrains, and its annual mean temperature is 9-12℃. It consists of the Tarim basin, dominated by the world's second largest sand desert (Taklamakan desert), and Dzungaria, an area mixed of desert, steppe and forest. Dzungaria and the Tarim basin are separated by the Tianshan mountains, a mountain range that bisects Xinjiang in half and forms part of the Inner Asian Mountain Corridor. The Tarim basin is dominated by the Taklamakan desert, which is characterized by high temperature and low rainfall, whereas the climate of Dzungaria is wetter and colder than that of the Tarim basin.

Xinjiang is surrounded by three high mountain ranges: the Altai mountains to the north that border Mongolia and Russia, the Kunlun Mountains to the south rising from the Tarim basin's southern edge to the Tibetan plateau, and the Pamir mountains to the west that wall off the Central Asia, namely Kazakhstan, Kyrgyzstan, Tajikistan, Afghanistan and Pakistan. Only in the east is Xinjiang topographically contiguous with other regions, primarily through the Hexi Corridor, which has long served to link Xinjiang to agropastoral communities farther east in the Gansu–Qinghai region and beyond. This geographic position has historically made Xinjiang a critical bridge connecting east and west Eurasia, most famously during the height of the "Silk Road" (114 BCE to 1450 CE). However, Xinjiang's role in facilitating cultural contact and the transmission of ideas across Eurasia can be traced much further back into prehistory[1]. Although there is growing evidence (e.g., surface stone tools) that humans may have inhabited the region as early as 40,000 BP at sites such as Tongtiandong and Jilintai in northern Xinjiang[2,3], the earliest human remains found thus far date only to the Early Bronze Age.

2. Archaeological sites and context

2.1 The Dzungaria Basin

The Dzungarian (Junggar) basin is located in north Xinjiang. At its center is the Gurbantunggut desert, which lies at an elevation of 200 meters above sea level (masl), while the surrounding mountains rise steeply to around 3,000 masl. The climate is hot and dry in summer and cold in winter, with an annual mean temperature of 5-8℃ in the lowlands and desert and 0-2 ℃ in the mountainous areas. The basin has relatively abundant water sources and annual precipitation of 300 mm in the mountains, but which can reach up to 600 mm in some places. Snow and ice melt feed rivers that flow into Dzungaria and support oases and large grasslands along the mountain foothills[4]. Large numbers of graves dating to the Bronze Age and later that resemble those of the nomadic cultures of the Eurasian steppe (e.g., Afanasievo and Chemurchek) have been discovered around the Gurbantunggut desert, and longstanding nomadic and transhumant pastoralist economies (sheep, goat, cattle and horse herding) in the region suggests that it has deep connections with prehistoric Eurasian steppe populations[4]. In this study, we analyzed individuals from three Dzungarian sites, each associated with Afanasievo culture: Ayituohan, Songshugou, and Nileke. These are currently the oldest human skeletons excavated in the Dzungarian Basin.

2.1.1 Ayituohan

The Ayituohan cemetery is located in Habahe County, in the far north of the Xinjiang Uyghur Autonomous Region of China. It was discovered and excavated by the Xinjiang

Institute of Cultural Relics and Archaeology in 2014 during the exploration of copper ores in the Ayituohan District of Habahe County. A total of 27 burials were excavated, and the individuals were dated to the first half of the third millennium BCE and are associated with the Afanasievo culture[5]. Two individuals (one male and a female) from the Ayituohan cemetery were subjected to shotgun sequencing and the male individual was directly radiocarbon dated (in bold). The female individual was dated based on the stratigraphy and archaeological contexts.

- AYIM22BY: **2843-2811 calBCE**
- AYIM22BN: 2800-2600 BCE

2.1.2 Songshugou

Songshugou cemetery was excavated in 2017 by a joint team from the Xinjiang Institute of Cultural Relics and Archaeology, the Jimunai Cultural Management office, and the Hoboksar Museum as part of a rescue excavation during the construction of the highway between Jimunai and Hoboksar County[6]. A total of 31 out of 71 burials along the highway were excavated and one sample from the Songshugou site was shotgun sequenced in this study. Associated material culture, such as egg-shaped vessels, indicate a close association with the Afanasievo culture.

- SSGM16: **2863-2801 calBCE**

2.1.3 Nileke

Nileke individuals were also excavated in 2017 by the Xinjiang Institute of Cultural Relics and Archaeology. A total of 112 individuals were excavated in the Nileke county. Four individuals were shotgun sequenced from the region, of which two yielded high-quality data available for downstream analyses. The Nileke individuals were identified as belonging to the Afanasievo culture based on the burial types and material culture, including 'egg-shaped' ceramics unique to the Afanasievo cuture[7].

- G218M5-2: **2907-2851 calBCE**
- G218M5-3N: **3005-2987 calBCE**

2.2 The Tarim Basin

The Tarim basin is located in south Xinjiang and enclosed by the Tianshan Mountains to the north, the Pamirs to the west and the Kunlun Mountains to the south. At its eastern end lies the former Salt Lake and marshland of Lop Nur. In the center of the basin is the Taklamakan desert, the second largest shifting sand desert in the world. The basin has an elevation of 780 meters above the sea level and the climate is extremely dry since the mountains block out moist air from the sea. The annual rainfall is less than 100 mm. All the above factors help to create the extremely arid desert climate in the Tarim Basin, which is now largely inhospitable[8]. However, rivers running down from the surrounding mountains support small oases around the perimeter of the Tarim Basin, and some rivers that run deep into the Taklamakan desert enable small-scale farming and animal herding in this otherwise very hostile environment. Due to the arid environment of the Tarim Basin, a wide diversity of organic materials has preserved there, including Buddhist Tocharian manuscripts (500-800 CE) found in the northern and eastern reaches of the Tarim Basin, organically well-preserved plant, animal, and even microbial (e.g., kefir) remains[9,10], and naturally mummified human remains, including the so-called "Beauty of Loulan", an Early Bronze Age mummy excavated at the site of Xiaohe in the eastern Lop Nur region of the Tarim Basin[11]. Recent archaeological studies in Xinjiang have found that several other cemeteries in the Tarim Basin, such as Gumugou near Xiaohe and Beifang in the Taklamakan hinterland, share common features (e.g. boat-shaped burials) with Xiaohe[12]. These sites have recently been

classified as belonging to the Xiaohe archaeological horizon. In this study, we analyzed individuals from three Tarim basin sites: Xiaohe, Gumugou, and Beifang[12,13,14].

2.2.1 Xiaohe

Xiaohe cemetery is located in the Lop Nur region of the Tarim Basin and was constructed on a salt-rich sand hill. The cemetery was discovered in 1910 by a local peasant, and later in 1934, the Swedish archaeologist Folke Bergman visited the cemetery and surveyed twelve graves[15]. The complete excavation of the Xiaohe cemetery was carried out by the Institute of Cultural Relics and Archaeology of Xinjiang between 2002 and 2005, during which a total of 167 graves were excavated. Five stratigraphic layers were identified spanning a period of 500 years. Two types of burials were encountered[12]. The majority of burials consisted of boat-shaped wooden coffins, that were without bottom but covered with wooden lids and cattle hides. Many of the human remains associated with this burial type were naturally mummified, and the graves also included well-preserved textiles and organic objects[9,16,17]. The second type of burial is consisting of wooden coffins covered with clay-lids[11]. This burial type was small in number (less than ten have been found so far) and was found only in the lowest (fourth and fifth) layers. In front of each coffin a wooden pole was erected, which for the female dead was frequently decorated with a round wooden post, while the male dead was frequently decorated by an oar-plank wooden post in front of the coffins. Many archaeologists considered that this may have a fertility significance. A wide array of burial goods have been excavated from the cemetery, including grass baskets (instead of pottery), arrows in male graves, and domesticated cereal grains of both eastern (e.g. millet) and western (e.g. wheat) Eurasian origins[17]. Up-to-date, archaeological reports for the uppermost three layers have been published, but until now there has been little publicly available on the older fourth and fifth layers. Cattle, goat and sheep remains were excavated from all five layers and it seems that Xiaohe people had a preference for cattle, as cattle were observed everywhere across the entire cemetery. For examples, only cattle hides were used to cover coffins, and the hearts of cattle were used to make cosmetics to paint the dead. Mummies with characteristic “European” cranial features were found across the five layers. All of the 11 samples analyzed in this study were from the fourth and fifth layers of Xiaohe cemetery. A total of 20 individuals were shotgun sequenced from Xiaohe, of which 11 yielded high-quality data.

- XHM100: **1884-1740 calBCE**
- XHM110: 2000-1800 BCE
- XHM135: **1936-1860 calBCE**
- XHM75: 2000-1800 BCE
- L5209: 2000-1800 BCE
- L5213: 2000-1800 BCE
- L4964: 2000-1800 BCE
- L6101: **1767-1623 calBCE**
- L6103: **1785-1664 calBCE**
- L6015: 2000-1800 BCE
- L6106: 2000-1800 BCE

An additional sample was screened for DNA but had insufficient preservation. A radiocarbon date was obtained for this sample.

- XHBM1: 2000-1800 BCE

Seven samples from Xiaohe were analyzed for proteomics, of which all seven yielded well-preserved proteomes.

- XHM100: **1884-1740 calBCE**
- XHM109: 2000-1800 BCE
- XHM112: 2000-1800 BCE
- XHM115: 2000-1800 BCE
- XHM117: 2000-1800 BCE
- XHM125: 2000-1800 BCE
  XHM135: **1936-1860 calBCE**

2.2.2 Gumugou

Like Xiaohe, the Gumugou Cemetery is also located in the Lop Nur region of the Tarim Basin. It was excavated in 1979 under the direction of Binghua Wang from the Institute of Cultural Relics and Archaeology of Xinjiang. A total of forty-two graves were excavated in the Gumugou cemetery, and two types of burials were identified: a sun-radiation-spokes burial pattern and the main pattern similar to the boat-shaped burials found in Xiaohe[12]. Only six burials belonged to the sun-radiation-spokes pattern, and based on the stratigraphic information, this pattern is slightly later than the boat-shaped burials. The sun-radiation-spoke burials are located in the northern part of the cemetery: at the center is a wooden rectangular coffin with a single male, which is surrounded by seven concentric ellipses made by wooden poles. Similar to Xiaohe, burial goods such as grass baskets with wheat and millet, cattle, sheep and goat as well as ephedra twigs were discovered. A total of three individuals were shotgun sequenced from Gumugou, of which one individual from a boat-shaped burial yielded high quality data.

- GMGM1: **1884-1736 calBCE**

2.2.3 Beifang

The Beifang cemetery is located in the heart of the Taklamakan desert and situated downstream from the ancient riverbed of the Keriya river. It is more than 500 kilometers southwest of the Xiaohe cemetery, and it is an even more isolated settlement than Xiaohe. The cemetery was discovered in 2008 and has features resembling those found at Xiaohe. The Beifang cemetery was also constructed on a mound and consists of boat-shaped coffins with oar-shaped posts erected at the head of male burials and multi-angled posts erected at the head of female burials. Some organically preserved goods such as woolen caps, wooden sculptures of human figures, woven baskets, and food made from wheat and millet as well as animal remains (e.g. cattle) were discovered[18]. The mummies excavated from the Beifang cemetery have a strong physical resemblance to the Xiaohe mummies, including fair-colored hair, long and high-bridged noses, and deep-set eyes[19]. A total of four individuals were shotgun sequenced from Beifang, of which one yielded high-quality data.

- 11KBM1: **1785-1664 calBCE**

3. Linguistic background of the population history in Xinjiang

Although many different languages through the ages have been spoken in the region, only one group is considered autochthonous, the Tocharian languages composed of Tocharian A and B. These languages are attested along the northern rim of the Tarim Basin during the middle of the first millennium CE. Using comparative methods, the common ancestor of these two languages, Proto-Tocharian, is estimated to have diverged at around 500 BC, presumably also in the northern Tarim Basin. By comparing its central vocabulary (e.g., numerals, pronouns, and central grammar), Proto-Tocharian reveals itself to be related to all

other Indo-European branches (incl. Greek, Latin, Germanic, Slavic, and Indo-Iranian) through their ancestral language Proto-Indo-European, which was spoken no later than 3000 BCE in the Pontic-Caspian Steppe. This suggests that Tocharian language branch experienced a significant eastward migration of its speakers during prehistory[20]. Parts of this prehistory may be gleaned from contacts with other languages. For example, a number of rather distinctive features of the Tocharian branch (including both sound and grammatical system) suggest extensive and intimate contacts with a Uralic language (likely related or similar to Finnish, Hungarian, Samoyedic, and several other minor languages in Russia[21,22]), while, on the other hand, certain Uralic lexical items (denoting, for example, numerals and metallurgy) appear to be of Tocharian provenance[23]. There are mutual borrowings between Proto-Tocharian and early Turkic (the predecessor of all modern Turkic languages, including Turkish, Chuvash, and Uighur), while Chinese elements within Tocharian[24] appear to postdate Tocharian borrowings into Chinese[25]. A small number of words has tentatively been associated with an extinct substrate language, possibly affiliated with the BMAC culture of Central Asia[26]. Indo-Iranian, the other Indo-European branch to migrate into Central Asia, similarly wielded influence on the Tocharian languages. Namely, their mainly lexical contributions are as complex as they are sustained, beginning with an unattested Old Iranian dialect already present in the Steppe Zone before Tocharian entered the Tarim Basin and continuing through the successive political, mercantile, and religious regimes of the Bactrians, Sogdians, Khotanese, and the Indic Prakrits that successively entered the region from the west[27].

**Language**	**Dating**	**Archaeological culture**	**Geography**	**Linguistic neighbors**
PIE	5500 BP	Yamnaya	Pontic-Caspian Steppe	-
Pre-Toch.	5000 BP<br>4500 BP<br>3000 BP	Afanasievo	Pontic-Caspian Steppe?<br>Minusinsk Basin?<br>Tarim Basin?	Uralic, Proto-Turkic<br>Old Chinese, Old Iranian
Proto-Toch.	2500 BP		Tarim Basin	Old Chinese
Toch.A&B	2500 BP		Tarim Basin	Middle Chinese, Old Uighur, Khotanese Saka (Iranian)

4. Detailed description of genetic isolation of the Tarim group

The Tarim_EMBA1 and Tarim_EMBA2 groups, although geographically separated by over 600 km of desert, cluster closely in PC space (Figs. 1-2) and show greatly reduced inter-individual genetic differences, as measured by extremely high outgroup-f_3 values and low pairwise mismatch rate (“pmr”) values of pseudo-diploid genotypes. The reduced genetic difference among the Tarim individuals is comparable to the level of 1st degree relatives in other published Bronze Age populations from the Eurasian steppe[28] (Extended Data Fig. 4A, C). However, a lack of long runs of homozygosity (ROH) segments expected for such close relatives suggests that a population bottleneck and not close kinship nor recent inbreeding is the likely explanation for the reduced genetic diversity (Extended Data Fig. 4B). Such observations were further supported by the fact that 12 out of 13 Tarim Basin individuals belong to a single mitochondrial haplogroup, C4 (Extended Data Table 1). Likewise,

although limited in number, the two Xiaohe males belong to the Y-chromosome haplogroup R1b1c, which falls outside of the R1b1a clade representative of the Yamnaya and Afanasievo individuals (Extended Data Table 1; Extended Fig. 4D; Supplementary Data S1B). The Y-chromosome of the Beifang male belongs to a basal R1 or R1b haplogroup but shares no derived allele with R1a or sublineage of R1b, similar to that of MA-1 (R*, xR1, xR2)[29] (Extended Data Table 1; Extended Fig. 4D).

5. Tarim mummies and the pre-pastoralist Central Asian genetic substratum

The Tarim mummies are among only a few known Holocene populations that derive the majority of their ancestry from Pleistocene ANE groups, who once made up the hunter-gatherer populations of southern Siberia, and which are represented by individual genomes from the archaeological sites of Mal'ta (MA-1)[29] and Afontova Gora (AG3)[30]. Interestingly, we observe that most Bronze Age and pre-Bronze Age populations with substantial ANE ancestry, such as Botai_CA from Eneolithic northern Kazakhstan, Kumsay_EBA and Mereke_MBA from western Kazakhstan, West_Siberia_N from Neolithic southern Russia, Okunevo_EMBA from the Minusinsk Basin, Chemurchek from EMBA Altai mountains, and Aigyrzhal_BA, Dali_EBA, Kanai_MBA from the IAMC region, show the highest outgroup-f_3 value with Tarim_EMBA1, suggesting that the Tarim mummies are currently the best representative of the pre-pastoralist ANE-related population that once inhabited Central Asia and southern Siberia (Extended Data Fig. 2A), even though Tarim_EMBA1 postdates these populations in time. This calls for a revision of previous admixture models of these populations, which were based on Botai_CA or West_Siberia_N[31,32,33], to include Tarim_EMBA1 as their ANE source (Supplementary Data S1F-H,I).

Applying qpAdm, we successfully modeled the high-ANE group West_Siberia_N as a mixture of Tarim_EMBA1 (67%) and Eastern European Hunter-Gatherers (EHG) (Supplementary Data S1I). Botai _CA shows a similar profile but requires an additional Eastern Eurasian contribution (5-12%) (Supplementary Data S1I; Extended Data Table 3). Chemurchek, Aigyrzhal_BA, and Dali_EBA fit to the three-way mixture of Tarim_EMBA1 (14-54%), Dzungaria_EBA1 (30-67%) and Geoksyur_EN (9-48%; a pre-BMAC proxy) while Chemurchek and Aigyrzhal_BA do not fit to Tarim_EMBA1+Afanasievo+Geoksyur_EN (Supplementary Data S1F-G). Mereke_MBA from western Kazakhstan also fit to Tarim_EMBA1+Dzungaria_EBA1 but not to Tarim_EMBA1+Afanasievo (Supplementary Data S1G). In contrast, Kumsay_EBA from western Kazakhstan fit to Tarim_EMBA1+Afanasievo+Geoksyur_EN but not to Tarim_EMBA1+Dzungaria_EBA1+Geoksyur_EN (Supplementary Data S1G; Extended Data Table 3). These results confirm that the genetic profile represented by the Tarim mummies is a good proxy for the ANE substratum that was once more widely distributed across pre-pastoralist Central Asia (Fig. 3C), and suggests that Dzungaria_EBA locally relayed Afanasievo ancestry into northern Xinjiang and its neighboring regions, and that IAMC/BMAC-related ancestry spread into Xinjiang independent of the dispersal of the Afanasievo herders. We find that the Chemurchek, an EBA pastoralist culture that succeeds

the Afanasievo in both the Dzungarian Basin and Altai mountains, derive from these three ancestry streams, which helps to explain both the IAMC/BMAC-related ancestry previously noted in Chemurchek individuals[31] as well as their reported cultural and genetic affiliations to Afanasievo groups[33]. Importantly, however, none of these admixed groups were the source population for the Tarim Basin Xiaohe culture, who we instead find were a highly isolated local autochthonous population. The extreme genetic bottleneck specific to Tarim_EMBA may have been related to the EMBA colonization of this challenging desert environment.

References

1. Jia, P. W., Betts, A., Cong, D., Jia, X. & Dupuy, P. D. Adunqiaolu: new evidence for the Andronovo in Xinjiang, China. *Antiquity* **91**, 621–639 (2017).
2. Yu, J. & He, J. Significant discoveries from the excavation of Jimunai Tongtiandong site, Xinjiang (in Chinese). *Cultural Relic of China* 8 (2017).
3. Ruan, Q. Studies on the discoveries of Andronovo affiliation found in Xinjiang, China (in Chinese). *Western Archaeology* 125–154 (2013).
4. Jia, P. W., Betts, A. & Wu, X. New evidence for Bronze Age agricultural settlements in the Zhunge'er (Junggar) Basin, China. *Journal of Field Archaeology* **36**, 269–280 (2011).
5. Xinjiang Institute of Cultural Relics and Archaeology. Archaeological report of the Ayituohan I cemetery in Habahe county, Xinjiang. *Xinjiang cultural relics* (2017).
6. Xinjiang Institute of Cultural Relics and Archaeology. Archaeological report of the Songshugou cemetery in the 219 national road near Hebusaier county, Xinjiang (in Chinese). *Cultural Relic of Xinjiang* 55–81 (2018).
7. Xinjiang Institute of Cultural Relics and Archaeology, The Xinjiang archaeological team of the Institute of Archaeology, Chinese Academy of Social Sciences, School of archaeology and Museology, Peking University, School of History, Renmin University & School of Cultural Relics, Northwest University. Archaeological findings in Xinjiang of 2017 (in Chinese). *Xiyu Yanjiu* (2018).
8. Zhang, Y. *et al.* Holocene environmental changes around Xiaohe Cemetery and its effects on human occupation, Xinjiang, China. *J. Geogr. Sci.* **27**, 752–768 (2017).
9. Yang, Y. *et al.* Proteomics evidence for kefir dairy in Early Bronze Age China. *Journal of Archaeological Science* **45**, 178–186 (2014).
10. Mai, H. *et al.* Characterization of cosmetic sticks at Xiaohe Cemetery in early Bronze Age Xinjiang, China. *Scientific Reports* **6**, 18939 (2016).
11. Wang, B. The Bronze Age cultures in peacock river region (in Chinese). *Science Press* 3–93 (2017).
12. Yang, Y. Shifting Memories: Burial Practices and Cultural Interaction in Bronze Age China: A study of the Xiaohe-Gumugou cemeteries in the Tarim Basin. (Uppsala University, 2019).
13. Chen, Y. & Zhang, Y. The mysterious Beifang cemetery (in Chinese). *Science and Culture* 22–23 (2008).
14. Wei, D. *et al.* Archaeological survey of the Loulan region during the Han and Jin dynasties- a joint cranimteric analysis of the Gutai cemetery in Loulan (in Chinese). *Xiyu Yanjiu* 126-132+172 (2020).
15. Abuduresule, I. Buried in the deep desert, how was Xiaohe cemetery discovered (in Chinese). *Xibu Kaifa* 146–151 (2019).
16. Rao, H. *et al.* Proteomic identification of adhesive on a bone sculpture-inlaid wooden artifact from the Xiaohe Cemetery, Xinjiang, China. *Journal of Archaeological Science* **53**, 148–155 (2015).
17. Qu, Y. *et al.* Diverse lifestyles and populations in the Xiaohe culture of the Lop Nur region, Xinjiang, China. *Archaeol Anthropol Sci* **10**, 2005–2014 (2018).
18. Xie, M. *et al.* Identification of a dairy product in the grass woven basket from Gumugou Cemetery (3800 BP, northwestern China). *Quaternary International* **426**, 158–165 (2016).
19. Mair, V. H. & Hickman, J. *Reconfiguring the Silk Road: New Research on East-West Exchange in Antiquity*. (University of Pennsylvania Press, 2014).
20. Fortson, B. W. *Indo-european language and culture: an introduction*. (Wiley-Blackwell, 2010).

21. Bednarczuk, L. Non-Indo-European features of the Tocharian dialects. in *Words and dictionaries. A Festschrift for Professor Stanisław Stachowski on the occasion of his 85th birthday* 55–67 (2015).
22. Peyrot, M. The deviant typological profile of the Tocharian branch of Indo-European may be due to Uralic substrate influence. *Indo-European Linguistics* **7**, 72–121 (2019).
23. Napol'skikh, V. Tocharisch-uralische Berührungen: Sprache und Archäologie. in *Early Contacts between Uralic and Indo-European: Linguistic and Archaeological Considerations* 367–383 (2001).
24. Lubotsky, A. & Srarostin, S. *Turkic and Chinese loan words in Tocharian*. (Mouton de Gruyter, 2003).
25. Lubotsky, A. Tocharian loan words in Old Chinese: Chariots, chariot gear, and town building. in *The Bronze Age and Early Iron Age Peoples of Eastern Central Asia* 379–390 (1998).
26. Carling, G. Proto-Tocharian, Common Tocharian, and Tocharian – on the value of linguistic connections in a reconstructed language. *Journal of Indo-European Monograph Series, No. 50* 48–71 (2005).
27. Tremblay, X. Irano-Tocharica et Tocharo-Iranica. *Bulletin of the School of Oriental and African Studies, University of London* **68**, 421–449 (2005).
28. Narasimhan, V. M. *et al.* The formation of human populations in South and Central Asia. *Science* **365**, eaat7487 (2019).
29. Raghavan, M. *et al.* Upper Palaeolithic Siberian genome reveals dual ancestry of Native Americans. *Nature* **505**, 87–91 (2014).
30. Fu, Q. *et al.* The genetic history of Ice Age Europe. *Nature* **534**, 200–205 (2016).
31. Jeong, C. *et al.* A Dynamic 6,000-Year Genetic History of Eurasia's Eastern Steppe. *Cell* **183**, 890–904 (2020).
32. Jeong, C. *et al.* The genetic history of admixture across inner Eurasia. *Nature Ecology & Evolution* **3**, 966–976 (2019).
33. Wang, C.-C. *et al.* Genomic insights into the formation of human populations in East Asia. *Nature* **591**, 413–419 (2021).
