# Supplementary material for: The genomic origins of the Bronze Age Tarim Basin mummies
Source: Nature. 2021 Oct 27;599(7884):256–61. doi: 10.1038/s41586-021-04052-7 (PMC8580821; doi:10.1038/s41586-021-04052-7)
Supplement: Supplementary file 2 — Reporting Summary [file 41586_2021_4052_MOESM2_ESM.pdf]

nature research

Corresponding author(s): Choongwon Jeong
Last updated by author(s): Aug 23, 2021

Reporting Summary

Nature Research wishes to improve the reproducibility of the work that we publish. This form provides structure for consistency and transparency in reporting. For further information on Nature Research policies, see our Editorial Policies and the Editorial Policy Checklist.

Statistics

For all statistical analyses, confirm that the following items are present in the figure legend, table legend, main text, or Methods section.

n/a	Confirmed	
☐	☒	The exact sample size (*n*) for each experimental group/condition, given as a discrete number and unit of measurement
☐	☒	A statement on whether measurements were taken from distinct samples or whether the same sample was measured repeatedly
☐	☒	The statistical test(s) used AND whether they are one- or two-sided<br>*Only common tests should be described solely by name; describe more complex techniques in the Methods section.*
☒	☐	A description of all covariates tested
☐	☒	A description of any assumptions or corrections, such as tests of normality and adjustment for multiple comparisons
☒	☐	A full description of the statistical parameters including central tendency (e.g. means) or other basic estimates (e.g. regression coefficient) AND variation (e.g. standard deviation) or associated estimates of uncertainty (e.g. confidence intervals)
☐	☒	For null hypothesis testing, the test statistic (e.g. *F*, *t*, *r*) with confidence intervals, effect sizes, degrees of freedom and *P* value noted<br>*Give P values as exact values whenever suitable.*
☒	☐	For Bayesian analysis, information on the choice of priors and Markov chain Monte Carlo settings
☒	☐	For hierarchical and complex designs, identification of the appropriate level for tests and full reporting of outcomes
☒	☐	Estimates of effect sizes (e.g. Cohen's *d*, Pearson's *r*), indicating how they were calculated

Our web collection on statistics for biologists contains articles on many of the points above.

Software and code

Policy information about availability of computer code

Data collection	Illumina sequence data were processed using the following programs to obtain genotype data used in the analysis: EAGER v1.92.55, AdapterRemoval v2.2.0, BWA v0.7.12, DeDup v0.12.2, bamUtils v1.0.13, mapDamage v2.0.9, pileupCaller v1.4.0.5 (https://github.com/stschiff/sequenceTools), Schmutzi v1.5.1, circularmapper v1.1, ANGSD v0.910. These programs are publicly available.
Data analysis	Calibration of AMS 14C dating results was done by OxCal v4.4, using the IntCal20 database. Population genetic data analysis in this study was performed using the following publicly available programs: lcMLkin v0.5.0, Geneious v11.1.3, HaploGrep2, bcftools v1.7, pysam v0.15.2, smartpca v16000, ADMIXTURE v1.3.0, PLINK v1.90, qp3Pop v435, qpDstat v755, hapROH v0.3a4 (https://pypi.org/project/hapROH/), DataGraph v4.5.1, qpWave v410, qpAdm v810, DATES v753. Non-default parameters used in our analysis are described in the Methods section. Protein mass spectrometry data analysis was performed using the following programs: MSConvert v3.0.11781, Mascot v2.6.0, Scaffold v4.9.0.

For manuscripts utilizing custom algorithms or software that are central to the research but not yet described in published literature, software must be made available to editors and reviewers. We strongly encourage code deposition in a community repository (e.g. GitHub). See the Nature Research guidelines for submitting code & software for further information.

Data

Policy information about availability of data

All manuscripts must include a data availability statement. This statement should provide the following information, where applicable:
- Accession codes, unique identifiers, or web links for publicly available datasets
- A list of figures that have associated raw data
- A description of any restrictions on data availability

The DNA sequences reported in this paper have been deposited in the European Nucleotide Archive (ENA) under accession PRJEB46875. Haploid genotype data of

ancient individuals in this study on the 1240k panel are available in the EIGENSTRAT format from the following link: https://edmond.mpdl.mpg.de/imeji/collection/OMm2fpu0jR3jSqnY. The protein spectra have been deposited in the ProteomeXchange Consortium via the PRIDE partner repository under accession PDX027706. The publicly available database SwissProt release 2019_08 is accessible through the UniProt Knowledge Base (https://www.uniprot.org). The basemaps used in Figs. 1 and 3 are in the public domain and accessible through the Natural Earth website (https://www.naturalearthdata.com/downloads/10m-raster-data/).

Field-specific reporting

Please select the one below that is the best fit for your research. If you are not sure, read the appropriate sections before making your selection.

☐ Life sciences ☐ Behavioural & social sciences ☒ Ecological, evolutionary & environmental sciences

For a reference copy of the document with all sections, see nature.com/documents/nr-reporting-summary-flat.pdf

Ecological, evolutionary & environmental sciences study design

All studies must disclose on these points even when the disclosure is negative.

Study description	This study includes whole genome or genome-wide sequencing of 18 Early/Middle Bronze Age ancient individuals from Xinjiang, out of 33 skeletal elements screened, ranging between 3000 and 1700 BC. Sequencing coverage ranges 0.06-1.44x. Ancient genomes come from both the Dzungarian basin (n=5) and the Tarim basin (n=13).
Research sample	Research samples are composed of 18 ancient genomes from various archaeological sites in Xinjiang. They are chosen to cover the archaeological cultures so far excavated in northern and southern Xinjiang. We separated them into four analysis units based on their geographic origin, time period, and individual genetic profiles. The analysis units include: Dzungaria_EBA1 (n=3), Dzungaria_EBA2 (n=2), Tarim_EMBA1 (n=12), Tarim_EMBA2 (n=1). Among the 18 ancient individuals, there are six genetic males and 12 genetic females. Individual genetic sex information is available at the Extended Data Table 1.
Sampling strategy	Sample size was determined by the availability of relevant archaeological specimen and therefore no pre-selection of sample size was performed prior to the study. To produce ancient genomes reported in this study, we screened the accessible skeletal elements from the relevant geographic regions and time periods, and produced in-depth sequencing data for those with sufficient endogenous DNA preservation and without substantial contamination.
Data collection	Yinqiu Cui and Qiaomei Fu were present for data collection. Libraries that were prepared in Jilin (n=26) were directly shotgun sequenced on an Illumina HiSeq X10 or HiSeq 4000 instrument at the Novogene company, China, in the 150-bp paired-end sequencing design. Libraries prepared at IVPP, samples with 0.1% or more human DNA from the initial sequencing (n=7) were enriched at approximately 1.2 million nuclear SNPs and were sequenced on an Illumina HiSeq 4000 instrument in Beijing, China using 2x150bp chemistry.
Timing and spatial scale	Laboratory works and sequencing were conducted over the period from March 2018 to June 2019. Samples were taken from various archaeological sites in Xinjiang, China. Detailed information of the archaeological samples studied in this manuscript is provided in Fig. 1 and Supplementary Data S1-v4.xlsx.
Data exclusions	We excluded samples only if the samples do not meet the quality criteria, either by having low level of endogenous human DNA prohibiting genome-scale sequencing or by showing high level of contamination estimates. For population genetic analysis that requires exclusion of genetic relatives, we excluded closely related individuals (1st degree relatives) by removing one with lower coverage from each pair.
Reproducibility	We took multiple individuals from each archaeological site, if available, to support the representativeness of their genetic and dental proteomic profiles. For each sample, we estimated contamination level to support the authenticity of data. Importantly, two labs produced genome data of different individuals from the Xiaohe site: Jilin (n=4) and IVPP (n=7). Repeated genome data collection on the same individual is not considered as necessary nor useful in the field standard and therefore was not performed.
Randomization	Ancient genomes were first analyzed by each individual, and then were allocated into the analysis group based on their archaeological context, absolute date (14C dating), and their individual genetic profile. Randomization is not applicable because this study is observational and includes no treatment nor case/control comparison.
Blinding	There was no experimental treatment of samples involved in this study that requires blinding. Data analysis was performed based on the analysis groups that were defined by external information (archaeological context and date).

Did the study involve field work? ☐ Yes ☒ No

Reporting for specific materials, systems and methods

We require information from authors about some types of materials, experimental systems and methods used in many studies. Here, indicate whether each material, system or method listed is relevant to your study. If you are not sure if a list item applies to your research, read the appropriate section before selecting a response.

Materials & experimental systems

n/a	Involved in the study
☒	☐ Antibodies
☒	☐ Eukaryotic cell lines
☐	☒ Palaeontology and archaeology
☒	☐ Animals and other organisms
☒	☐ Human research participants
☒	☐ Clinical data
☒	☐ Dual use research of concern

Methods

n/a	Involved in the study
☒	☐ ChIP-seq
☒	☐ Flow cytometry
☒	☐ MRI-based neuroimaging

Palaeontology and Archaeology

Specimen provenance

The archaeological human remains studied in this manuscript were excavated by the Xinjiang Institute of Cultural Relics and Archaeology during 1979-2017. Scientific investigation of these remains were approved by the Xinjiang Cultural Relics and Archaeology Institute, which holds the custodianship of the studied remains, based on the written agreements.

Specimen deposition

The archaeological human remains studied in this manuscript are being housed in and managed by the Xinjiang Institute of Cultural Relics and Archaeology.

Dating methods

Of the 18 individuals reported this study, 10 were directly dated using accelerator mass spectrometry (AMS) at Beta Analytic, Miami, USA and/or at Lanzhou University, China. To confirm the reliability of our AMS datings, 4 out of the 10 individuals were AMS dated at both Beta Analytic and Lanzhou University. Consistent dates were obtained in all cases (Supplementary Data S1C). The calibration of the dated samples were performed based on the IntCal20 database and using the OxCal v4.4 program.

☒ Tick this box to confirm that the raw and calibrated dates are available in the paper or in Supplementary Information.

Ethics oversight

This study is based on previously excavated archaeological remains and included no new excavation effort nor study of live human or animal subjects. Therefore the study protocols used in this study are not the subject of approval by IRB/IACUC. The access to the remains was approved by the Xinjiang Institute of Cultural Relics and Archaeology based on the written agreements.

Note that full information on the approval of the study protocol must also be provided in the manuscript.
